# Supplementary material for: Trip Centrality: walking on a temporal multiplex with non-instantaneous link travel time
Source: Sci Rep. 2019 Jul 22;9:10570. doi: 10.1038/s41598-019-47115-6 (PMC6646307; doi:10.1038/s41598-019-47115-6)
Supplement: Supplementary file 1 — Supplementary Info [file 41598_2019_47115_MOESM1_ESM.pdf]

Supplementary Information
Trip Centrality: walking on a temporal multiplex with non-instantaneous link travel time

Silvia Zaoli[1], Piero Mazzarisi[1], and Fabrizio Lillo[1]

[1]Dipartimento di Matematica, University of Bologna, Bologna, Italy

1 Computing Trip Centrality when $\varepsilon = 1$

When $\varepsilon = 1$, the matrix K used in the computation of Trip Centrality (equation (4) of the main text) is not invertible, therefore Trip Centrality cannot be computed using equation (4). In this section, we prove that, if the final multiplication by K^{-1} in equation (4) is skipped, the rankings according to the single-layer outgoing centrality and the aggregated outgoing and incoming centralities do not change (although the values of the centralities do change). We conclude, therefore, that the rankings according to these centralities for the case $\varepsilon = 1$, computed skipping such final multiplication, can be safely compared with the rankings obtained with equation (4) for other values of ε. Consider Trip Centrality, and let

$$M = [(\mathbb{I} + \tilde{\alpha} A^{[1]} K) \dots (\mathbb{I} + \tilde{\alpha} A^{[T]} K) - \mathbb{I}] K^{-1}. \tag{1}$$

The element M_{ij} contains the sum of the weights of walks of all lengths from i to j. The incoming Trip Centrality of the node j is then obtained as $t_j^{in} = \sum_i M_{ij}$ and the outgoing Trip Centrality of the node i as $t_i^{out} = \sum_j M_{ij}$.
Let $\tilde{M}$ be the equivalent of M but skipping the final division by K,

$$\tilde{M} = [(\mathbb{I} + \tilde{\alpha} A^{[1]} K) \dots (\mathbb{I} + \tilde{\alpha} A^{[T]} K) - \mathbb{I}]. \tag{2}$$

The element $\tilde{M}_{ij}$ counts, on top of the walks counted by M_{ij}, also the walks that arrived to one of the copies of j on other layers and then jumped to j in the last step, with an additional weight ε due to the final change of layer. Calling C_j the set of copies of j, we have thus

$$\tilde{M}_{ij} = M_{ij} + \varepsilon \sum_{k \in C_j \setminus \{j\}} M_{ik} = (1 - \varepsilon) M_{ij} + \varepsilon \sum_{k \in C_j} M_{ik}. \tag{3}$$

Note that, if j is a secondary node, $C_j = \{j\}$, and $\tilde{M}_{ij} = M_{ij}$. The single-layer centralities computed from $\tilde{M}$ are, therefore:

$$\tilde{t}_j^{in} = \sum_i \tilde{M}_{ij} = (1-\varepsilon)t_j^{in} + \varepsilon \sum_i \sum_{k \in C_j} M_{ik} = (1-\varepsilon)t_j^{in} + \varepsilon \sum_{k \in C_j} t_k^{in}, \tag{4}$$

$$\begin{aligned} \tilde{t}_i^{out} &= \sum_j \tilde{M}_{ij} = (1-\varepsilon)t_i^{out} + \varepsilon \sum_j \sum_{k \in C_j} M_{ik} = (1-\varepsilon)t_i^{out} + \varepsilon N_L t_i^{out} \\ &= (1+(N_L-1)\varepsilon)t_i^{out}, \end{aligned} \tag{5}$$

where N_L is the number of layers.
In the outgoing case, t and $\tilde{t}$ differ only by a multiplicative constant, therefore multipliying or not by K^{-1} produces the same ranking. This is not true, however, for the incoming case (for single-layer centrality).
Let us now check what happens for the aggregated centralities of primary nodes.

$$\tilde{t}_j^{in,a} = \sum_{k \in C_j} \tilde{t}_k^{in} = \sum_{k \in C_j} [(1-\varepsilon)t_k^{in} + \varepsilon \sum_{l \in C_k} t_l^{in}] = (1-\varepsilon)t_j^{in,a} + \varepsilon N_L t_j^{in,a} = (1+(N_L-1)\varepsilon)t_j^{in,a}, \tag{6}$$

$$\tilde{t}_i^{out,a} = \sum_{k \in C_i} \tilde{t}_k^{out} = \sum_{k \in C_i} (1+(N_L-1)\varepsilon)t_k^{out} = (1+(N_L-1)\varepsilon)t_i^{out,a}. \tag{7}$$

We obtain that, for both the incoming and the outgoing case, the aggregated centrality with or without multiplication by K^{-1} differ only by a multiplicative constant, therefore produce the same ranking.

2 Computational complexity

In this section, we give an upper bound to the computational complexity of the algorithm needed to compute Trip Centrality. The computation of the matrix Q in eq. (2) of the main text consists in T products of matrices of size $(N+N_l)\times(N+N_l)$, which are typically very sparse. If the products are computed starting from the leftmost factor, at each step we perform a matrix multiplication of the type $Q^{[t+1]} = Q^{[t]}(\mathbb{I} + \alpha A^{[t+1]})$, where $Q^{[t]} = (\mathbb{I} + \alpha A^{[1]}) \dots (\mathbb{I} + \alpha A^{[t]})$. This product has the form

$$\left[\begin{array}{c|c} A & B \\ \hline C & D \end{array}\right] \times \left[\begin{array}{c|c} \mathbb{I}_{N\times N} & E \\ \hline F & \mathbb{I}_{N_l\times N_l} \end{array}\right] = \left[\begin{array}{c|c} A+BF & B+AE \\ \hline C+DF & D+CE \end{array}\right], \tag{8}$$

where A is an $N\times N$ matrix, B and E are $N\times N_l$, C and F are $N_l\times N$ and D is $N_l\times N_l$. The matrices E and F contain the stubs linking primary nodes to secondary ones and viceversa during that time frame. In each time frame, on average, there will be N_l/T non-zero entry in each of the two matrices, because N_l is the total number of links. For A, B, C and D, as an upper bound, we assume that all entries are non-zero. Let us then count the number of operations (multiplications and additions) needed to compute the

block $A + BF$. If F was not sparse, the product BF would require $N^2 N_l$ operations. However, for each of the N rows of B that I multiply by the N columns of F, on average only $N_l/(TN)$ elements will be non-zero. Therefore, by using an appropriate algorithm for sparse matrices, which does not perform operations on the null entries, the number of operations required is only $N \cdot N \cdot N_l/(TN) = NN_l/T$. Therefore, computing $A+BF$ requires $N^2 + NN_l/T$ operations. With similar reasoning on the other three blocks, and considering that a similar matrix multiplication must be performed T times, the total number of operations required to compute Q is $2NN_l + 2N_l^2 + T(N + N_l)^2$. Therefore, the computational complexity is $O(T(N + N_l)^2)$. Furthermore, if the network is sparse, i.e. $N_l = O(N)$, the complexity becomes $O(N^2)$.

3 TripRank: a generalisation of PageRank to temporal multiplexes

PageRank is a generalisation of Katz centrality, developed by Google [1], that introduces an additional weight to the walks, depending on the in- (or out-) degree of the nodes they cross. Considering a static directed network of N nodes with weighted adjacency matrix A_{ij}, such that $A_{ij} = k$ if there are k links from i to j. The incoming PageRank centrality of node i is given by the sum of the contributions of walks of any length incoming to i, where each walk of length n contributes α^n times a factor $1/d_l^{out}$ for each node l crossed by the walk, with d_l^{out} the out-degree of node l. This sum can be computed as

$$pr_i^{in} = \sum_{n=0}^{\infty} \sum_{j=1}^{N} (\alpha D^{-1} A)_{ji}^n = \sum_{j=1}^{N} (\mathbb{I} - \alpha D^{-1} A)_{ji}^{-1}, \tag{9}$$

where $D_{ij} = \delta_{ij} d_i^{out}$ with δ_{ij} the Kronecker delta, so that using a link from j to k is weighted by the inverse of the out-degree of j, $1/d_j^{out}$. Convergence requires α to be smaller than the maximum eigenvalue of $D^{-1}A$. In terms of our air traffic example, the meaning of this generalization is that an airport with an inbound flight coming from a large airport, with a large out-degree, will inherit a fraction of its centrality proportional to the inverse of such out-degree. In other words, the more outbound flights an airport has, the less of its centrality such destination airport inherits. In the out going case, similarly we have

$$pr_i^{out} = \sum_{j=1}^{N} (\mathbb{I} - \alpha A D^{-1})_{ji}^{-1}, \tag{10}$$

where $D_{ij} = \delta_{ij} d_i^{in}$. Convergence requires α to be smaller than the maximum eigenvalue of AD^{-1}.

The single-layer temporal generalisation of PageRank, here termed *Single-Layer*

TripRank, is obtained analogously to Single-Layer Trip Centrality as

$$\vec{tr}_S^{out} = [(\mathbb{I} + \tilde{\alpha} A^{[1]} D_{in}^{-1})(\mathbb{I} + \tilde{\alpha} A^{[2]} D_{in}^{-1}) \dots (\mathbb{I} + \tilde{\alpha} A^{[T]} D_{in}^{-1}) - \mathbb{I}]\vec{1}_{N+N_l}, \tag{11a}$$

$$\vec{tr}_S^{in} = \vec{1}_{N+N_l}^T [(\mathbb{I} + \tilde{\alpha} D_{out}^{-1} A^{[1]})(\mathbb{I} + \tilde{\alpha} D_{out}^{-1} A^{[2]}) \dots (\mathbb{I} + \tilde{\alpha} D_{out}^{-1} A^{[T]}) - \mathbb{I}], \tag{11b}$$

where the temporal adjacency matrices $A^{[t]}$ are defined as in the main text and D_{in} and D_{out} are the diagonal matrices $D_{in,ij} = \delta_{ij} d_i^{in}$ and $D_{out,ij} = \delta_{ij} d_i^{out}$, with d_i^{in} and d_i^{out}, respectively, the number of incoming and outgoing links of node i during the entire observation window.
The temporal multiplex generalisation, named TripRank, is also obtained analogously to Trip Centrality:

$$\vec{tr}^{out} = [(\mathbb{I} + \tilde{\alpha} A^{[1]} D_{in}^{-1} K)(\mathbb{I} + \tilde{\alpha} A^{[2]} D_{in}^{-1} K) \dots (\mathbb{I} + \tilde{\alpha} A^{[T]} D_{in}^{-1} K) - \mathbb{I}] K^{-1} \vec{1}_{NN_L+N_l}, \tag{12a}$$

$$\vec{tr}_S^{in} = \vec{1}_{NN_L+N_l}^T [(\mathbb{I} + \tilde{\alpha} D_{out}^{-1} A^{[1]} K)(\mathbb{I} + \tilde{\alpha} D_{out}^{-1} A^{[2]} K) \dots (\mathbb{I} + \tilde{\alpha} D_{out}^{-1} A^{[T]} K) - \mathbb{I}], \tag{12b}$$

where the matrix K and the multilayer adjacency matrices $A^{[t]}$ are defined as in the main text and D_{in} and D_{out} are the diagonal matrices $D_{in,ij} = \delta_{ij} \tilde{d}_i^{in}$ and $D_{out,ij} = \delta_{ij} \tilde{d}_i^{out}$, with $\tilde{d}_i^{in}$ and $\tilde{d}_i^{out}$, respectively, the number of incoming and outgoing links of node i or any of its copies on the other layers during the entire observation window.

4 Eliminating walks that pass a second time from the starting point

The elimination of walks that pass a second time from their starting point is obtained as follows. Let

$$Q = (\mathbb{I} + \tilde{\alpha} A^{[1]} K) \dots (\mathbb{I} + \tilde{\alpha} A^{[T]} K). \tag{13}$$

With this definition,

$$\vec{t}^{out} = [Q - \mathbb{I}] K^{-1} \vec{1}_{NN_L+N_l}, \tag{14a}$$

$$\vec{t}^{in} = \vec{1}_{NN_L+N_l}^T [Q - \mathbb{I}] K^{-1}. \tag{14b}$$

The element Q_{ik} contains the contribution to the outgoing centrality of i given by walks from i to j (or equivalently the contribution to the incoming centrality of j given by walks from i to j). If we compute the matrix Q one time frame at a time, $Q^{[1]} = (\mathbb{I} + \tilde{\alpha} A^{[1]} K)$ only counts the walks during the first time frame, $Q^{[2]} = (\mathbb{I} + \tilde{\alpha} A^{[1]} K)(\mathbb{I} + \tilde{\alpha} A^{[2]} K)$ counts the walks up to the second time frame, and so on. At each time step, the diagonal elements have value $Q_{ii}^{[t]} = 1 +$ contribution of walks going from i to i. By setting the diagonal elements to 1 after each multiplication, we eliminate the contributions of walks coming back to their starting point during that time frame. This eliminates also all the walks which would be the continuation of such loops.

5 Results of US air traffic datasets analysis with TripRank

5.1 Comparison between Trip Centrality and TripRank

The rankings of airports according to Trip Centrality and TripRank are quite different: with $\varepsilon = 0$ and $\alpha = 0.2$, they have a Kendall correlation coefficient, averaged over all days in the dataset, $\tau = 0.70 \pm 0.01$ in the incoming case and $\tau = 0.69 \pm 0.02$ in the outgoing case. Increasing ε the correlation decreases further. TripRank penalises long walks more than Trip Centrality does, as each additional flight brings a weight α/d, with d the degree of its departure or arrival airport (depending on whether we consider incoming or outgoing TripRank), typically much smaller than α. Therefore, it is not surprising that increasing ε, which means that more long walks are considered and increasingly weighted, decreases the similarity of the two metrics. For the same reason, the correlation coefficient of the two metrics increases if TripRank is computed with a larger α, for example for $\varepsilon = 0$ and $\alpha = 0.8$ for TripRank, we find $\tau = 0.76 \pm 0.01$ in the incoming case and $\tau = 0.74 \pm 0.01$ in the outgoing one. Supplementary Fig. S8a shows the comparison of the rankings for April 1st, with $\varepsilon = 0$ and $\alpha = 0.2$, in the incoming case. It can be seen that the larger differences are given by airports gaining a large number of ranks with TripRank. Similar results are observed for the other days in the dataset. The airports gaining many ranks with TripRank are typically the ones that are connected to airports with a very small degree, e.g. the airport of Anchorage, with flights to other small regional airports in Alaska. In fact, these kind of connections in the case of TripRank assure a relatively high centrality contribution, while according to Trip Centrality they give the same contribution as a connection to a large airport, which however brings additional centrality through longer walks, favouring airports connected to large airports. Increasing the value of α used to compute TripRank reduces these differences (see supplementary Fig. S8b), however the rankings remain fundamentally different.

5.1.1 Comparison of TripRank and PageRank

In this section, we compare the ranking of airports according to TripRank and to its static single-layer counterpart, PageRank. PageRank is computed with the largest value of α allowing convergence, $\alpha = 0.19$. The Kendall correlation coefficient between the rankings according to the two metrics computed using the same value of α is $\tau = 0.80 \pm 0.02$ in the incoming case and $\tau = 0.79 \pm 0.02$ in the outgoing case when $\varepsilon = 0$, and grows with increasing ε, as expected given that PageRank does not distinguish layers (see supplementary Fig. S11). A comparison of the rankings for April 1st, for the incoming case, is shown in supplementary Fig. S10. For ε close to 1, the two ranking become quite similar (Fig. S11b). Results are qualitatively similar for other days of the dataset. The correlations increase very slightly for increasing values of α used in the computation of TripRank. The top ten airports according to the two centrality metrics

are reported in the supplementary table 7.

5.2 Comparison of airports' ranking for different values of ε

Figure S9 shows how the airports' ranking according to outgoing and incoming TripRank on the scheduled network on April 1st changes when $\varepsilon = 0, 0.1, 0.2, 0.3, 0.5, 0.8$. Results for other days of April are qualitatively similar. Differently from what was shown in the main text for Trip Centrality, in this case the most central airports are quite stable when ε varies, and in general rank increases are smaller than with Trip Centrality. This is explained by the fact that the percentage variations of centrality values caused by changes of ε are much smaller than those found with Trip Centrality, as long walks are more penalised by TripRank, and are therefore unable to change the ranking of the most central airports, which have large centrality gaps.

6 Elimination of new walks created by delays in the realised network

When computing Trip Centrality metrics in the realised network, new walks that were not present in the scheduled network appear due to the changes in the link schedules (i.e., in the air traffic case, delays). As explained the Methods section of the main text, in the air traffic case these walks should be excluded from the computation, as passengers cannot use them. On the contrary, for other transportation systems, e.g. bus and metro, itineraries do not need to be fixed in advance, therefore the new walks can be used.
In this section, we show how we can exclude from the computation the new walks appearing when the delay of a departing flight allows the connection with an incoming flight, which was originally landing too late to make the connection.
Consider Trip Centrality, and let

$$Q = (\mathbb{I} + \tilde{\alpha} A^{[1]} K) \dots (\mathbb{I} + \tilde{\alpha} A^{[T]} K). \tag{15}$$

With this definition,

$$\vec{t}^{out} = [Q - \mathbb{I}] K^{-1} \vec{1}_{NN_L + N_l}, \tag{16a}$$

$$\vec{t}^{in} = \vec{1}^{T}_{NN_L + N_l} [Q - \mathbb{I}] K^{-1}. \tag{16b}$$

The element Q_{ij} contains the contribution to the outgoing centrality of i given by walks from i to j (or equivalently the contribution to the incoming centrality of j given by walks from i to j). We call Q the matrix computed with A_{sched}, and Q_r the matrix computed with A_{real} . Now, let us compute the matrix Q one time frame at a time. $Q^{[1]} = (\mathbb{I} + A^{[1]} K)$ only counts the walks during the first time frame, $Q^{[2]} = (\mathbb{I} + A^{[1]} K)(\mathbb{I} + A^{[2]} K)$ counts the walks up to the second time frame, and so on. At each time step, if an element of Q_r is larger than the corresponding element of Q_s,

it is because of a new walk opened up by a delay. In fact, in the real network departure and landings take place either at the same time or later than in the scheduled network (having put all negative delays to zero, as motivated in the Methods section), therefore all new acceptable contributions to centrality are added to Q_r either at the same time as in Q_s, or later. Therefore, at each step we pose $Q_r = \min\{Q_s, Q_r\}$. This eliminates most spurious walks. The correction procedure is analogous for TripRank, with the appropriate definition of Q.

In order to better understand the principle of this correction, let us consider a concrete example. Let i, j and k be three primary nodes, and let there be two flights from i to j, f1 departing at $t = 1$ and landing at $t = 2$, and f2 departing at $t = 3$ and landing at $t = 4$. Additionally, assume there is a flight from j to k, called f3, departing at $t = 3$ and landing at $t = 4$. According to schedule, it is possible to go from i to k with f1+f3, but not with f2+f3. However, assume now that f3 is delayed, and departs at $t = 5$, arriving at $t = 6$. In the realised network, it would now be possible to reach j from i also with f2+f3. However, the proposed correction eliminates this contribution. In fact, calling a the secondary node associated with flight f3, we have $Q^{[5]}_{s,ia} = \tilde{\alpha}^3$, which counts the walk given by f1 and f3, and $Q^{[5]}_{r,ia} = 2\tilde{\alpha}^3$, which counts also the walk given by f2+f3. Now, taking $Q^{[5]}_{r,ia} = \min\{Q^{[5]}_{r,ia}, Q^{[5]}_{s,ia}\} = \tilde{\alpha}^3$, the combination f2+f3 is correctly removed. At the following timestep, then, we get $Q^{[6]}_{s,ik} = Q^{[6]}_{r,ik} = \tilde{\alpha}^4$.

We remark that there are still some 'forbidden' walks that remain, even after this correction, however they are very rare. Specifically, if in the example above f1 was delayed such that the combination f1+f3 became impossible, while the delay of f3 still makes the combination f2+f3 possible in the realised network, in the realised network we should obtain that $Q^{[6]}_{r,ik} = 0$, as one walk is impossible and the other one is forbidden. However, at $t = 5$ we have $Q^{[5]}_{s,ia} = \tilde{\alpha}^3$, which counts the walk given by f1 and f3, and $Q^{[5]}_{r,ia} = \tilde{\alpha}^3$, which counts only the walk given by f2+f3. Then, at the following time step we would get $Q^{[6]}_{s,ik} = Q^{[6]}_{r,ik} = \tilde{\alpha}^4$. Therefore, in this case, a walk of two flights is still counted in the realised network, although it is forbidden. However, the realisation of this situation requires an improbable combination of delays. For example, on April 1st there are only 142 such combinations, on a total of almost 2 millions triplets of flights that could potentially give rise to such combinations. Additionally, 82 of these 142 are inter-layer, therefore not counted when $\varepsilon = 0$. We conclude that the number of forbidden walks remaining after the correction introduced in this section is so small that they give a negligible contribution to centrality and therefore do not invalidate the comparison between the scheduled and realised networks.

7 Supplementary tables and figures

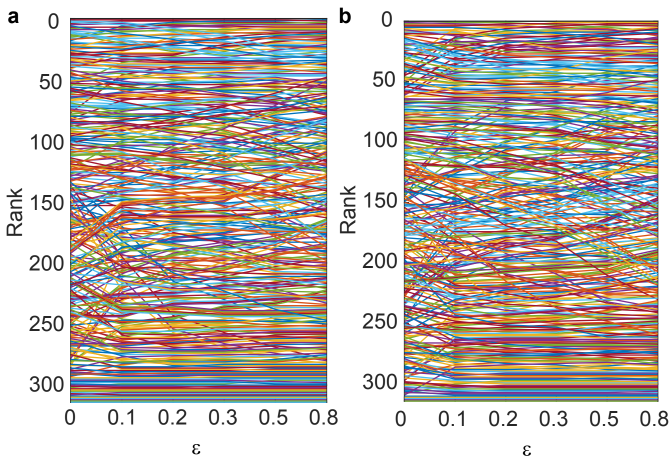

Figure S1: Evolution of the airports' ranking according to outgoing (a) and incoming (b) Trip Centrality on the scheduled network on April 1st for $\alpha = 0.2$ and different values of ε. Each line represents one airport, and the position on the y-axis indicates its rank for each ε value. Rank 1 corresponds to the most central airport.

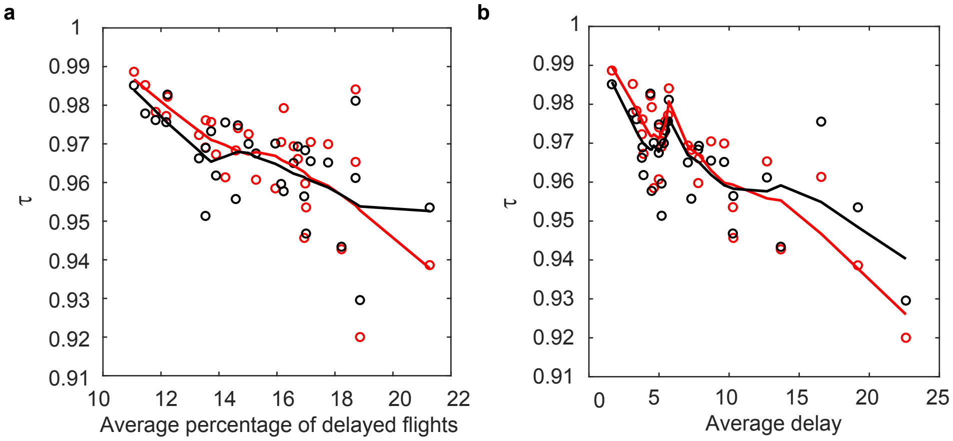

Figure S2: Kendall correlation coefficient between the airports' ranking on the scheduled and realised network, in each day of the dataset, according to incoming Trip Centrality (red) and outgoing Trip Centrality (black), plotted against the average fraction of flights with departure delay in one airport (a) and (b) the average departure delay (in minutes). Each point corresponds to one day of the dataset. Lines are obtained by a locally weighted smoothing (LOWESS) of the dots of the correspondent color.

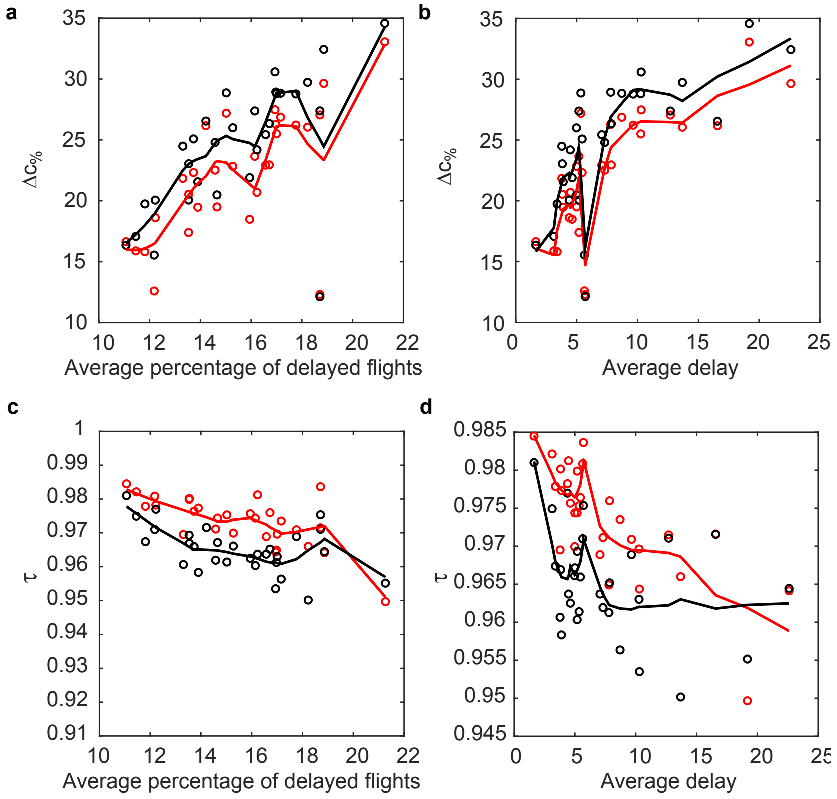

Figure S3: Replica of the analysis in the main text, excluding cancelled and diverted flights. a and b: Percentage of centrality loss, averaged over all airports, in each day of the dataset, plotted against the average percentage of delayed flights in an airport (a) and the average delay of flights (b), according to incoming Trip Centrality (red circles) and outgoing Trip Centrality (black circles). Trip centrality is computed with $\alpha = 0.2$ and $\varepsilon = 0$. The percentage centrality loss of an airport is computed as $\Delta c_{\%} = 100 \times (c_{sched} - c_{act})/c_{sched}$, where c_{sched} and c_{act} are the airport's centralities on the scheduled and realized network. Lines are obtained by a locally weighted smoothing (LOWESS) of the circles of the correspondent color. c and d: Kendall correlation coefficient between the airports' ranking on the scheduled and realised network plotted against the average percentage of delayed flights in an airport (c) and the average delay of flights (d), according to incoming Trip Centrality (red circles) and outgoing Trip Centrality (black circles). Lines are obtained by a locally weighted smoothing (LOWESS) of the circles of the correspondent color.

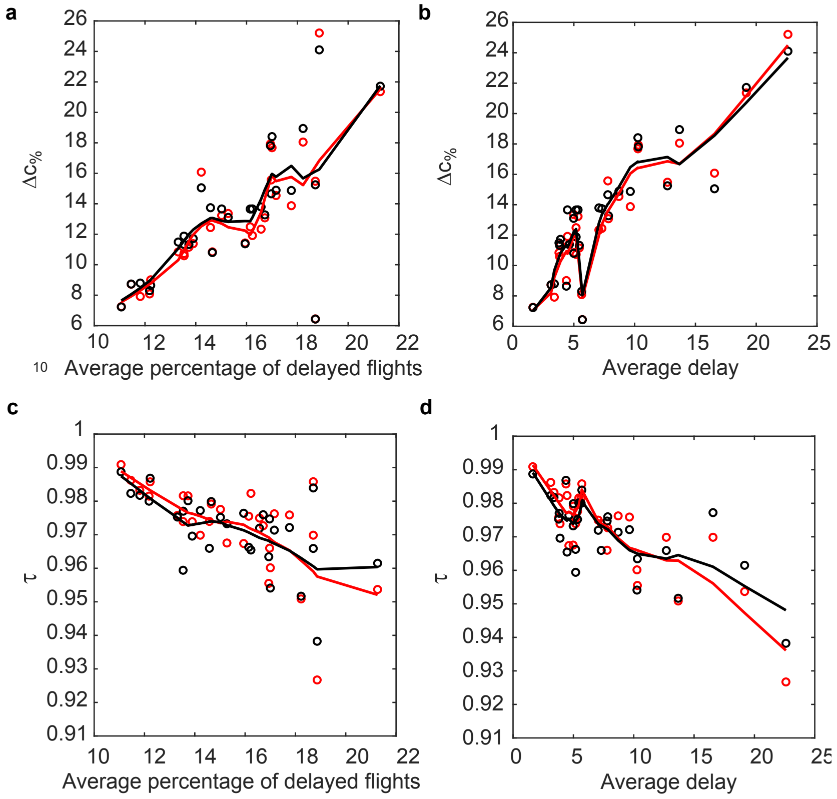

Figure S4: Replica of the analysis in the main text with $\alpha = 0.1$. a and b: Percentage of centrality loss, averaged over all airports, in each day of the dataset, plotted against the average percentage of delayed flights in an airport (a) and the average delay of flights (b), according to incoming Trip Centrality (red circles) and outgoing Trip Centrality (black circles). The percentage centrality loss of an airport is computed as $\delta c_{\%} = 100 \times (c_{sched} - c_{act})/c_{sched}$, where c_{sched} and c_{act} are the airport's centralities on the scheduled and realized network. Lines are obtained by a locally weighted smoothing (LOWESS) of the circles of the correspondent color. c and d: Kendall correlation coefficient between the airports' ranking on the scheduled and realised network plotted against the average percentage of delayed flights in an airport (c) and the average delay of flights (d), according to incoming Trip Centrality (red circles) and outgoing Trip Centrality (black circles). Lines are obtained by a locally weighted smoothing (LOWESS) of the circles of the correspondent color.

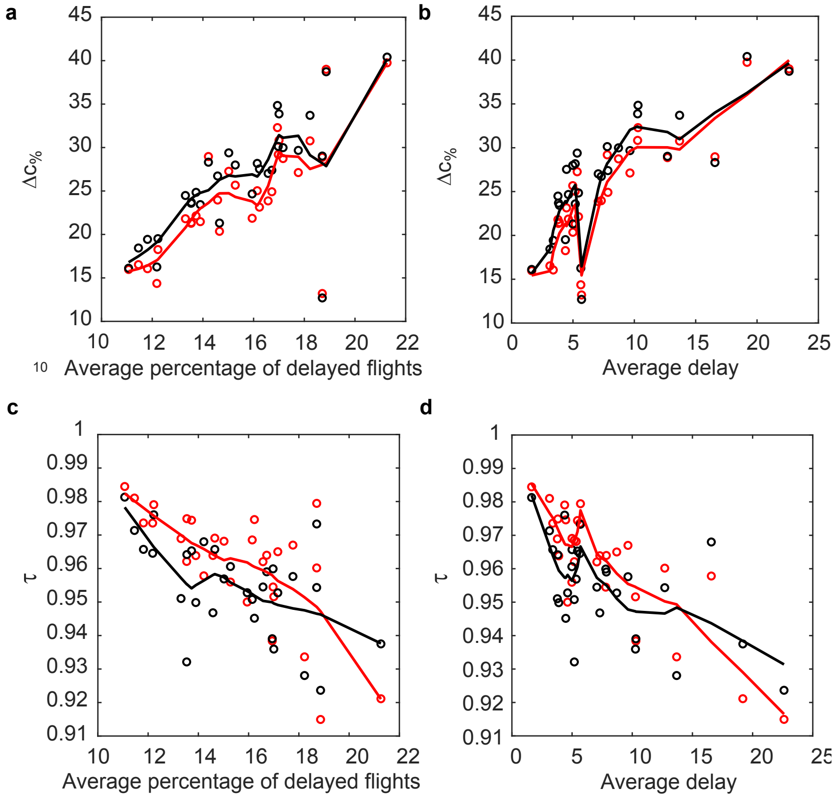

Figure S5: Replica of the analysis in the main text with $\alpha = 0.5$. a and b: Percentage of centrality loss, averaged over all airports, in each day of the dataset, plotted against the average percentage of delayed flights in an airport (a) and the average delay of flights (b), according to incoming Trip Centrality (red circles) and outgoing Trip Centrality (black circles). The percentage centrality loss of an airport is computed as $\delta c_{\%} = 100 \times (c_{sched} - c_{act})/c_{sched}$, where c_{sched} and c_{act} are the airport's centralities on the scheduled and realized network. Lines are obtained by a locally weighted smoothing (LOWESS) of the circles of the correspondent color. c and d: Kendall correlation coefficient between the airports' ranking on the scheduled and realised network plotted against the average percentage of delayed flights in an airport (c) and the average delay of flights (d), according to incoming Trip Centrality (red circles) and outgoing Trip Centrality (black circles). Lines are obtained by a locally weighted smoothing (LOWESS) of the circles of the correspondent color.

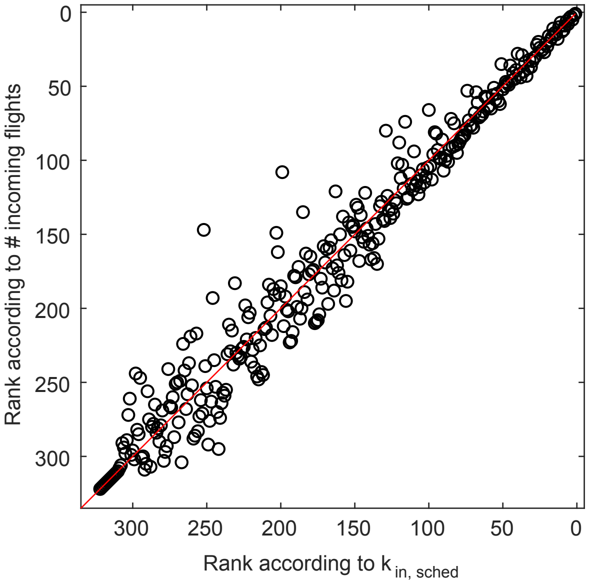

Figure S6: Comparison of the ranking according to incoming Katz centrality with $\alpha = 0.003$ and the ranking according to the number of incoming flights for April 1st, on the scheduled network.

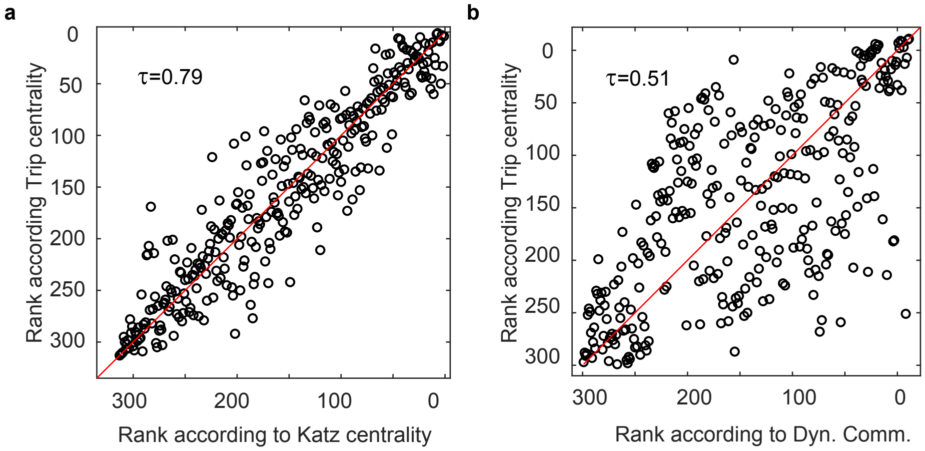

Figure S7: Comparison of airports' rankings on April 1st according to a) incoming Katz centrality and Trip Centrality and b) incoming Dynamic communicability and Trip Centrality. Trip Centrality is computed with $\alpha = 0.2$ and $\varepsilon = 0$. Each circle represents an airport, rank 1 corresponds to the highest centrality. The red line is the 1:1 line. The Kendall correlation coefficients are reported in the figure.

**Katz centrality**	**Trip Centrality**	**Dynamic communicability**
Atlanta	Phoenix	Chicago O'Hare
Chicago O'Hare	Las Vegas McCarran	New York (JFK)
Los Angeles	Los Angeles	Phoenix
Dallas/Fort Worth	Atlanta	Boston Logan
Denver	San Diego	Miami
San Francisco	Oakland	Charlotte Douglas
Phoenix	Sacramento	Denver
Las Vegas McCarran	San Francisco	Philadelphia
LaGuardia	Denver	Los Angeles
Boston Logan	Orlando	Newark

Table S1: Top ten airports on April 1st according to incoming Katz Centrality, Trip Centrality and Dynamic communicability. Katz centrality is computed with $\alpha = 0.003$, Trip Centrality and Dynamic communicability with $\alpha = 0.2$, Trip Centrality is computed with $\varepsilon = 0$.

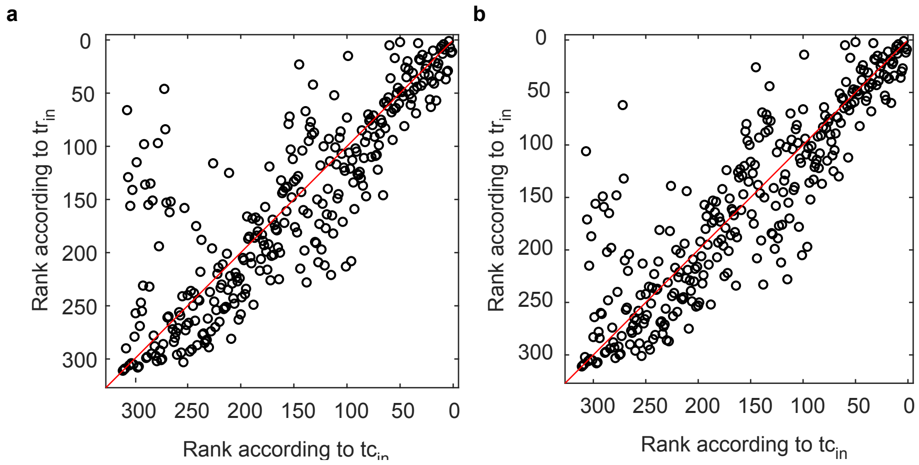

Figure S8: Comparison of the rankings according to incoming Trip Centrality and to TripRank for April 1st, on the scheduled network and with $\varepsilon = 0$. In panel a), both metrics are computed with $\alpha = 0.2$, in panel b) TripRank is computed with $\alpha = 0.5$. The red line is the 1:1 line.

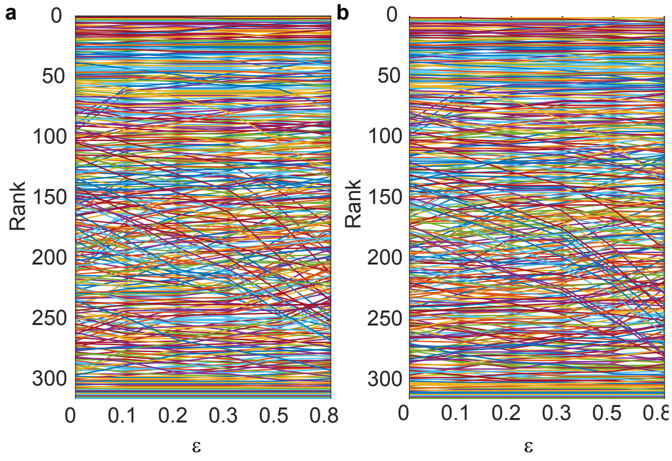

Figure S9: Evolution of the airports' ranking according to outgoing (a) and incoming (b) TripRank on the scheduled network on April 1st for different values of ε. TripRank is computed with $\alpha = 0.5$. Each line represents one airport, and the position on the y-axis indicates its rank for each ε value. Rank 1 corresponds to the most central airport.

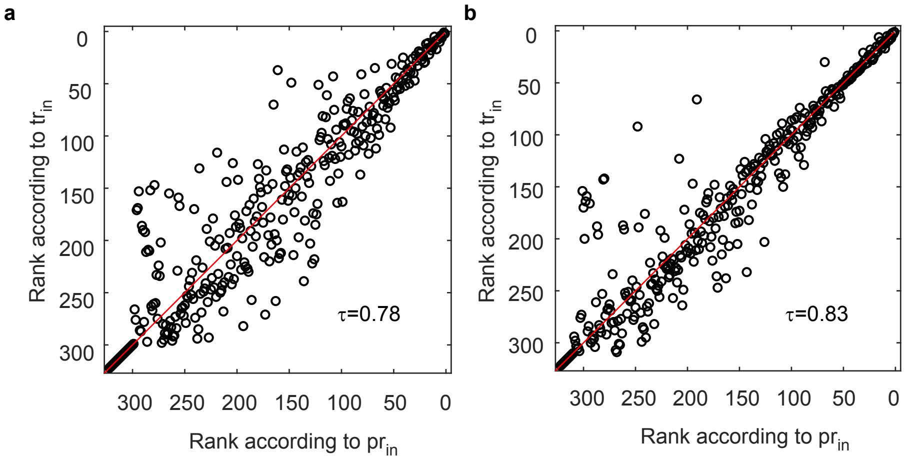

Figure S10: Comparison of the rankings according to incoming TripRank and PageRank for April 1st, on the scheduled network. TripRank is computed with $\alpha = 0.2$ and with $\varepsilon = 0$ in panel a) and $\varepsilon = 0.8$ in panel b). The red line is the 1:1 line.

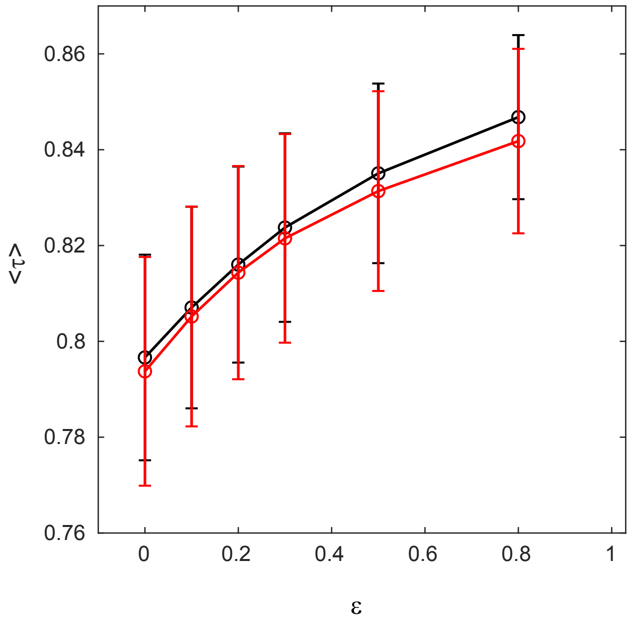

Figure S11: Kendall correlation coefficient, averaged on all days, between the rankings according to TripRank and Page Rank, for different values of ε used in the computation of TripRank. TripRank is computed with $\alpha = 0.2$. The red line corresponds to the incoming centralities and the black one to the outgoing. Error bars represent standard errors.

**PageRank**	**TripRank** ($\varepsilon = 0$)	**TripRank** ($\varepsilon = 0.3$)
Atlanta	Atlanta	Atlanta
Chicago O'Hare	Dallas/Fort Worth	Dallas/Fort Worth
Dallas/Fort Worth	Chicago O'Hare	Chicago O'Hare
Los Angeles	Denver	Denver
Denver	George Bush	George Bush
Phoenix	Salt Lake City	Salt Lake City
San Francisco	Los Angeles	Los Angeles
George Bush	Minneapolis-Saint Paul	San Francisco
Las Vegas McCarran	San Francisco	Minneapolis-Saint Paul
Boston Logan	Detroit	Detroit

Table S2: Top ten airports on April 1st according to incoming PageRank and TripRank. TripRank is computed with $\alpha = 0.2$.

References

[1] Brin, S. & Page, L. The anatomy of a large-scale hypertextual web search engine. *Comput. Netw. ISDN Syst.* **30**, 107–117 (1998).
